# Suppression of inflammation by helminths: a role for the gut microbiota?

**DOI:** 10.1098/rstb.2014.0296

**Published:** 2015-08-19

**Authors:** Paul Giacomin, John Croese, Lutz Krause, Alex Loukas, Cinzia Cantacessi

**Affiliations:** 1 Centre for Biodiscovery and Molecular Development of Therapeutics, Australian Institute of Tropical Health and Medicine, James Cook University, Smithfield 4878, Australia; 2 Department of Gastroenterology and Hepatology, The Prince Charles Hospital, Brisbane 4007, Australia; 3 Translational Research Institute, University of Queensland Diamantina Institute, Woolloongabba, Australia; 4 Department of Veterinary Medicine, University of Cambridge, Cambridge CB3 0ES, UK

**Keywords:** microbiota, host–parasite interactions, helminth-induced suppression of inflammation, hookworms, whipworms, microbial richness

## Abstract

Multiple recent investigations have highlighted the promise of helminth-based therapies for the treatment of inflammatory disorders of the intestinal tract of humans, including inflammatory bowel disease and coeliac disease. However, the mechanisms by which helminths regulate immune responses, leading to the amelioration of symptoms of chronic inflammation are unknown. Given the pivotal roles of the intestinal microbiota in the pathogenesis of these disorders, it has been hypothesized that helminth-induced modifications of the gut commensal flora may be responsible for the therapeutic properties of gastrointestinal parasites. In this article, we review recent progress in the elucidation of host–parasite–microbiota interactions in both animal models of chronic inflammation and humans, and provide a working hypothesis of the role of the gut microbiota in helminth-induced suppression of inflammation.

## 1. Introduction

The human gastrointestinal tract is inhabited by approximately 10^13^–10^14^ bacterial cells, which together are known as the gut microbiota. This complex network of commensal microorganisms exerts a number of specialized functions beneficial to the host, including absorption of nutrients, synthesis of essential organic compounds, protection against pathogens and contribution to the development of the intestinal immune system [1,2]. Perturbations of the gut microbial ecology (= ‘dysbiosis’) have been implicated in a number of diseases, including obesity, malnutrition, type I and type II diabetes, cancer and neurological disorders [1,3]. In addition, intestinal dysbiosis is associated with a range of chronic inflammatory disorders of the gastrointestinal tract, including Crohn's disease (CD), ulcerative colitis (UC) [4] and coeliac disease (CeD) [5]. These diseases exact an enormous toll in developed countries, with CD and UC being the two most common forms of inflammatory bowel disease (IBD), estimated to cost the economy of the United Kingdom alone approximately £1 billion per year [6].

CD and UC are lifelong inflammatory conditions of the colon and small intestine, characterized by aberrant responses of the mucosal immune system against the commensal flora [7]. While genetic factors contribute to the susceptibility to IBD [7], a number of studies support a pivotal role for environmental factors such as the gut microbiota in the pathogenesis of these chronic conditions [8]. IBD is associated with alterations in the nature of the microbial communities within the gut, which may affect immune development and intestinal barrier function. As a result, the breakdown in tolerance and compartmentalization of commensal microorganisms then perpetuates disease by stimulating the activation of inflammatory T cells, resulting in chronic inflammation [8]. While the exact mechanisms which determine this cascade of biological events are yet to be fully determined, a high concentration of mucosally-associated bacteria, together with the presence of enteric bacterial pathogens (e.g. adherent/invasive *Escherichia coli* and enterotoxigenic *Bacteroides fragilis*) and host factors contributing to intestinal dysbiosis (e.g. impaired bacterial killing) have been hypothesized to contribute to the development and severity of disease [8].

A role for intestinal dysbiosis in the pathogenesis and severity of CeD has been recently hypothesized [5,9]. CeD is an autoimmune disorder caused by an inappropriate response to dietary gluten, where symptoms include intestinal pain and discomfort, chronic constipation or diarrhoea, impaired nutrient absorption, anaemia and fatigue [10]. In people with CeD, ingestion of even trace amounts of gluten (10–50 mg), a major component of foods containing wheat, barley and rye, can cause infiltration of pro-inflammatory T cells to the small intestine, which causes apoptosis in the epithelial cells that form the intestinal barrier [11]. This inflammation is compounded by the production of autoantibodies against enzymes that process gluten, which are deposited in the intestine and promote truncation of the villi lining and destruction of the epithelial barrier [12]. An expansion in populations of *Bacteroides* spp., together with a reduction of *Bifidobacterium* spp., has been associated with the development of CeD [13]. Interestingly, adherence to a strict gluten-free diet (GFD) does not result in restoration of microbial balance, thus leading to the hypothesis of a link between genotype and intestinal dysbiosis that may predispose to disease [9]. To date, there are no effective cures for IBD or CeD that will enable affected individuals to engage in normal diets and lead symptom-free lives; however, modulation of the gut microbiota via the use of prebiotics, probiotics, antibiotics or faecal transplants could be viable therapeutic strategies.

## 2. Helminth-therapy to treat inflammatory gut disorders

**Figure 1.**
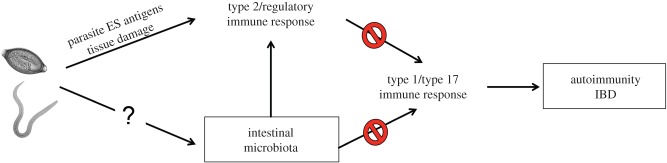
Potential role for microbiota in helminth-mediated suppression of autoimmune diseases? Helminths, including *Trichuris* sp. and hookworms are thought to limit the severity of IBDs and autoimmune diseases via promotion of type 2 and regulatory T cell responses that counteract pro-inflammatory type 1 or type 17 immune responses. However, emerging evidence suggests that helminth-mediated immune modulation may be, in part, due to alterations in the composition of the intestinal microbiota, which can profoundly influence immune cell development and function in the intestine. ES, excretory/secretory. (Online version in colour.)

One theory for the increased incidence of allergic and autoimmune diseases in the developed world, including IBD and CeD, is that improved sanitation has reduced our exposure to pathogens in childhood, which affects the development of the immune system. Consequently, there are increases in the incidence of immune disorders related to inappropriate responses to harmless stimuli—commonly referred to as the ‘hygiene hypothesis’ [14]. Therefore, in recent years, there have been multiple attempts to exploit the hygiene hypothesis via the controlled re-introduction of infectious agents with immunosuppressive properties, such as parasitic helminths [15–18]. In particular, two gastrointestinal parasitic nematodes, namely whipworms (*Trichuris* sp.) and hookworms (*Necator americanus*), have been investigated in a range of studies in both humans and animal models aimed at developing novel treatment strategies against IBD and CeD, respectively [16,19]. As a consequence of these pilot studies, there have been intriguing observations that some of the immunoregulatory capacity of worms may be directly or indirectly related to alterations in intestinal microbial communities (figure 1), which will be the focus of the remainder of this article.

## 3. Therapeutic potential of helminths and the role of the gut microbiota

### (a) Whipworms

Whipworms of the genus *Trichuris* are parasites of the large intestine of mammals. Infection occurs following the ingestion of the embryonated eggs, which hatch in the small intestine and release the infective larvae that develop to adults within the large intestine, partially embedding themselves within the epithelial lining [20,21]. Interestingly, the presence of commensal bacteria within the host is essential for the hatching of *Trichuris* eggs and hence the successful establishment of *Trichuris* infection [22]. Indeed, using individual *in vitro* cultures of five strains of bacteria (including *E. coli*, an extremely common gut commensal) and one of yeast, Hayes *et al*. [22] observed successful hatching of embryonated eggs of the murine whipworm, *T. muris*, similarly to that induced by incubation of eggs with tissue explants of mouse caecum containing the commensal microflora. Removal of bacterial or yeast cells from cultures prevented hatching, thus revealing, for the first time, a close association between a metazoan parasite and the gut microbiota [22]. Like many other species of intestinal helminths, *Trichuris* infection elicits a biased type 2 immune response in its host, which is thought to be involved in downregulating type 1 and type 17 immune responses that are typically associated with many autoimmune diseases including IBD [16].

Of the approximate 70 known *Trichuris* species, *T. trichiura* and *T. suis* are known as the human and porcine whipworms, respectively. However, the latter can establish asymptomatic, transient infections in the large intestine of humans [23], thus providing scope for investigations into the role of this parasite as a safe alternative therapeutic strategy for allergic and autoimmune disorders [23]. Indeed, in recent years, several studies have reported amelioration of clinical symptoms of CD and UC in patients subjected to oral administration of *T. suis* ova (TSO) [16,24–26]. In addition, ingestion of TSO by volunteers affected by multiple sclerosis was followed by a reduction in number of brain lesions (as verified by magnetic resonance imaging), thus providing evidence for the systemic nature of the immune response elicited by this parasite [27].

Efforts to elucidate the mechanisms by which experimental whipworm infections lead to improvement of clinical symptoms of immune-mediated diseases have primarily focused on the immunoregulatory properties of parasites [16]. For instance, a number of studies using mouse models of IBD and helminth infections [28] have demonstrated that elements of both the innate and adaptive immune systems are modulated by helminths. These include suppression of interferon (IFN)γ and interleukin (IL)-17 expression, increased type 2 cytokine responses, induction of regulatory T cell responses and cytokines such as IL-10, transforming growth factor (TGF)-beta and IL-22 and the recruitment of alternatively activated macrophages, dendritic cells and B cells [28,29]. However, the exact mechanisms by which administration of TSO results in improvement of clinical indices of inflammation in human patients affected by IBD remain to be elucidated. Importantly, while regulation of the host immune system by helminths such as TSO is one of the likely mechanisms by which the parasites can suppress IBD symptoms, whether these effects are direct (via the excretion of immunomodulatory parasite proteins) or indirect, for example via alterations in the nature of intestinal environment (e.g. the microbiota) remains to be defined.

Given the pivotal roles that disturbances in the intestinal microbiota play in multiple immune disorders [1], and the fact that gastrointestinal parasites and the commensal flora share the same environmental niche [30], there is an increasing interest in understanding helminth–microbiota interactions and their relative contributions to health and disease. For instance, studies involving experimental infections with *Heligmosomoides polygyrus bakeri* in a mouse model of IBD revealed a significant expansion of the bacterial family Lactobacillaceae in the ileum of infected mice, which correlated with improved disease outcome [31,32]. Similarly, the administration of a single dose of TSO was able to alter the composition of the gut microbiota of infected pigs, including a reduction in the abundance of *Fibrobacter* and *Ruminococcus* and an expansion of *Campylobacter* [33]. In addition, a study using a primate model of idiopathic chronic diarrhoea (ICD) has demonstrated that the therapeutic ability of *T. trichiura* whipworms to improve clinical symptoms of inflammation was associated with significant changes in the composition and relative abundance of different gut bacterial species [34]. In particular, a marked reduction in the bacterial phylum Cyanobacteria was observed following *Trichuris* administration to macaques with ICD when compared with healthy controls, accompanied by an expansion of Bacteroidetes and Tenericutes; in addition, bacterial diversity was increased in *Trichuris*-infected ICD macaques [34]. The authors attributed these changes to the restoration of a ‘healthy’ flora driven by the parasite [34]. Notably, bacterial attachment (one of the key factors contributing to the pathogenesis of IBD) was substantially reduced following *Trichuris* treatment, thus suggesting a role for the parasite in mucosal healing which, in turn, results in a reduction of bacteria-mediated immune-stimulation [34,35]. Each of these studies investigating the relationships between helminth parasites and the commensal flora in animal models of inflammation have shed at least some light on the potential roles of the gut microbiota in whipworm-mediated suppression of inflammation [36]. However, in order to establish whether similar mechanisms occur during helminth infections in humans, studies of the impact of parasite colonization on the composition of the human gut microbiota are necessary.

### (b) Hookworms

Hookworms, including *Ancylostoma duodenale* and *N. americanus*, are blood-feeding nematodes that inhabit the small intestine of humans [18]. *Necator americanus* is the most widely distributed human hookworm, causing significant morbidity for the infected host when worm burdens become high and/or diet is inadequate [37]. The infection occurs when the infective third-stage larvae (L3s) penetrate the skin of a susceptible human host after cuticular shedding [38]; subsequently, larvae enter the subcutaneous tissue and migrate to the small intestine, via the circulatory system, to the heart and lungs, where they moult to fourth stage larvae (L4s). From the lungs, the larvae migrate (via the trachea and pharynx) to the small intestine, where they develop to adult males and females [38]. The larvae mature to adult stages and attach by their buccal capsule to the intestinal mucosa where their voracious appetite for blood results in iron-deficiency anaemia, the major pathogenesis associated with hookworm infection.

While heavy burdens of hookworm parasites are associated with pathological effects, experimental infections with small numbers of *N. americanus* are safe and well tolerated [39–42]. In addition, the chronic nature of hookworm infections presents advantages for helminth-based therapy when compared with administration of *T. suis*—as the parasite is adapted to long-term survival in humans it need not be continuously administered. In modern sanitary environments, hookworm-infected individuals pose no risk of transmission to others [15]. The immune response to hookworm infection is similar to that of other intestinal helminths (including whipworms), with increased expression of the regulatory cytokines IL-10 and TGFβ, expansion of Foxp3+ regulatory T cells, increased IL-22 and IL-5 expression, and reductions in IL-23, IFNγ and IL-17A levels [42–44]. In addition, when administered to mouse models of IBD, hookworm excretory/secretory products protect against inflammation and weight loss [45], thus providing support to further investigations of hookworm-based therapies for the treatment of chronic gut inflammatory diseases of humans. Indeed, experimental *N. americanus* infections have been shown to confer temporary benefit to patients with active CD [46], and improve gluten tolerance in CeD volunteers [43]. However, similarly to *Trichuris*-based therapies, the elucidation of the exact mechanisms by which these parasites are able to suppress the symptoms of chronic inflammation is pivotal to the transfer of hookworm-based therapies from the laboratory to the clinic.

Recently, our laboratory conducted a pilot study to explore the impact of experimental infections with *N. americanus* on the human gut microbiota [47]. Eight volunteers were infected with *N. americanus* larvae while on GFD, and the changes in relative abundance of individual bacterial species within the faeces were analysed [47]. Following massively parallel sequencing of two distinct hypervariable regions of the bacterial 16S rRNA gene and bioinformatics analyses of sequence data, Principal Coordinates Analysis revealed strong clustering of the samples by individual, rather than by infection status, thus indicating that the community composition of each subject remained stable over time [47]. Interestingly, an increase in the number of observed bacterial species (= richness) was observed eight weeks post-infection; however, this difference was just below the statistical significance level when corrected for multiple testing [47]. Further investigations are currently underway examining the changes in commensal bacterial communities at later time points and following gluten challenges.

This observation of increased bacterial richness supports the results from a recent study [48] showing that human infections by gastrointestinal helminths (including *Trichuris*, hookworms and the intestinal roundworm *Ascaris* sp.) in endemic areas are associated with an increase in richness and diversity of the gut microbiota. Intriguingly, a higher species richness of the gut microbiota has been associated with ‘healthier’ intestinal homeostasis [49–51]. For example, a study comparing the intestinal microbiota of subjects suffering from IBD with that of healthy controls revealed that species richness was significantly higher in the latter [49]; in addition, the microbiota isolated from ‘histologically normal’, non-inflamed tissue from diseased subjects displayed a significantly increased species richness when compared with that from inflamed (as assessed by histological examination) biopsy samples from the same individuals [49]. Therefore, based on the results of our and others' investigations linking an increase in microbial species richness to (i) human infections by gastrointestinal parasites [47,48] and (ii) the absence or amelioration of clinical and histological indices of inflammation [34,49–51], it is tempting to speculate that the therapeutic properties of hookworms and other helminths are partly associated with their ability to promote species richness and restore/maintain microbial (and immune) homeostasis in the gastrointestinal tract [47]. Clearly, in order to address this hypothesis in greater detail, larger human trials in a variety of inflammatory disease settings are required, where both faecal and mucosally-associated bacterial communities at the sites of inflammation are examined.

## 4. Concluding remarks

The complex relationships between the human host and the commensal gut microbiota have been hypothesized to result from a lengthy process of coevolution, whereby the host benefits from the metabolic functions of the gut microbes while providing them with a protective environment [52]. Gastrointestinal parasitic helminths have also evolved numerous strategies to survive and reproduce within the body of the host [53]; therefore, it is conceivable that, in a dysbiotic environment, parasites may actively (directly or indirectly) contribute to reinstating the gut homeostasis via modulating the composition of the gut microbiota. Indeed, over the last few years, a body of evidence has been generated that links infections by gastrointestinal parasitic helminths with quantitative and qualitative modifications of the gut microbiota, in both animals and humans, and under both natural and experimental settings [31,34,47,48,53,54]. However, the nature of the parasite–microbiota interactions that underpin such modifications is yet to be elucidated. In a recent study, Reynolds *et al*. [32] hypothesized that changes in the composition of the gut microbiota of mice infected with *H. polygyrus* may be a consequence of: (i) the secretion of antimicrobial components by the parasite that actively modify the microbiota, (ii) the disruption of the epithelial barrier by the parasite that alters the intestinal environment and favours the establishment of selected commensals, or (iii) the stimulation of specific immune responses (such as expansion of Tregs) that actively contribute to a shift in gut microbiota [32]. Whether parasite-associated changes in gut microbiota are a direct consequence of the infection or, rather, the immune response elicited by helminths remains to be determined. In the future, mechanistic studies, in both normal and germ-free mice, focusing on the effects of experimental infections with gastrointestinal helminths on both the gut microbiota and host responses will facilitate elucidation of this intriguing conundrum. For instance, studies of global gene expression changes occurring at the site of parasite attachment (and, for instance, in response to the administration of an inflammatory stimulus) may provide a better understanding of the relationships between the parasite, the host responses and the ecology of the gut microbiota and may, in turn, assist the development of novel, helminth-based therapeutics of chronic inflammatory gut disorders.
